# Nanopore Single-Molecule Sequencing for Mitochondrial DNA Methylation Analysis: Investigating Parkin-Associated Parkinsonism as a Proof of Concept

**DOI:** 10.3389/fnagi.2021.713084

**Published:** 2021-09-28

**Authors:** Theresa Lüth, Kobi Wasner, Christine Klein, Susen Schaake, Ronnie Tse, Sandro L. Pereira, Joshua Laß, Lasse Sinkkonen, Anne Grünewald, Joanne Trinh

**Affiliations:** ^1^Institute of Neurogenetics BMF, University of Lübeck and University Hospital Schleswig-Holstein Campus Lübeck, Lübeck, Germany; ^2^Luxembourg Centre for Systems Biomedicine, University of Luxembourg, Belvaux, Luxembourg; ^3^Department of Life Sciences and Medicine, University of Luxembourg, Belvaux, Luxembourg

**Keywords:** mitochondrial DNA, methylation, Nanopore, third-generation sequencing, Parkinson's disease, Parkin

## Abstract

**Objective:** To establish a workflow for mitochondrial DNA (mtDNA) CpG methylation using Nanopore whole-genome sequencing and perform first pilot experiments on affected *Parkin* biallelic mutation carriers (Parkin-PD) and healthy controls.

**Background:** Mitochondria, including mtDNA, are established key players in Parkinson's disease (PD) pathogenesis. Mutations in Parkin, essential for degradation of damaged mitochondria, cause early-onset PD. However, mtDNA methylation and its implication in PD is understudied. Herein, we establish a workflow using Nanopore sequencing to directly detect mtDNA CpG methylation and compare mtDNA methylation between Parkin-related PD and healthy individuals.

**Methods:** To obtain mtDNA, whole-genome Nanopore sequencing was performed on blood-derived from five Parkin-PD and three control subjects. In addition, induced pluripotent stem cell (iPSC)-derived midbrain neurons from four of these patients with PD and the three control subjects were investigated. The workflow was validated, using methylated and unmethylated 897 bp synthetic DNA samples at different dilution ratios (0, 50, 100% methylation) and mtDNA without methylation. MtDNA CpG methylation frequency (MF) was detected using Nanopolish and Megalodon.

**Results:** Across all blood-derived samples, we obtained a mean coverage of 250.3X (SD ± 80.5X) and across all neuron-derived samples 830X (SD ± 465X) of the mitochondrial genome. We detected overall low-level CpG methylation from the blood-derived DNA (mean MF ± SD = 0.029 ± 0.041) and neuron-derived DNA (mean MF ± SD = 0.019 ± 0.035). Validation of the workflow, using synthetic DNA samples showed that highly methylated DNA molecules were prone to lower Guppy Phred quality scores and thereby more likely to fail Guppy base-calling. CpG methylation in blood- and neuron-derived DNA was significantly lower in Parkin-PD compared to controls (Mann-Whitney U-test *p* < 0.05).

**Conclusion:** Nanopore sequencing is a useful method to investigate mtDNA methylation architecture, including Guppy-failed reads is of importance when investigating highly methylated sites. We present a mtDNA methylation workflow and suggest methylation variability across different tissues and between Parkin-PD patients and controls as an initial model to investigate.

## Introduction

Given the importance of environmental impact on mtDNA and its association with disease, the investigation of mtDNA CpG methylation is of interest. For example, nuclear genome methylation correlates with age (Benayoun et al., 2015; Jiang and Guo, 2020), and it has been suggested that mtDNA can behave similarly, as an association between age and methylation level of two CpG sites within mtDNA has been observed (Mawlood et al., 2016).

The most common method to detect mtDNA methylation has been bisulfite treatment. With bisulfite treatment, the characterization of the methylation patterns depends on the resistance of 5-methylcytosine (5-mC) to be converted to uracil. However, the secondary and tertiary structure of intact mtDNA may block cytosines from bisulfite conversion, which influences the outcome of the detected methylation levels (Mechta et al., 2017; Owa et al., 2018). Bisulfite conversion-resistant cytosines can lead to an overestimation of mtDNA methylation. To counteract the bias of bisulfite-resistant cytosines, linearization of mtDNA has been recommended (Owa et al., 2018). For linearized mtDNA prior to bisulfite treatment, lower levels of methylation have been observed compared to intact mtDNA (Liu et al., 2016; Mechta et al., 2017). The low levels of methylation have led to suggestions of this being artifactual, suggesting an absence of methylation in the mitochondrial genome altogether (Hong et al., 2013; Mechta et al., 2017). By contrast, there has also been supportive evidence for the presence of mtDNA methylation after linearization (Sun et al., 2018; Patil et al., 2019). Furthermore, cytosines outside of the CpG context have been reported to be relatively highly methylated (Bellizzi et al., 2013; Patil et al., 2019). The level of mtDNA methylation and its biological significance is still under debate. In the specific case of Parkinson's disease (PD), one study has shown altered methylation in the non-coding D-loop region of patient mtDNA (Blanch et al., 2016). In the D-loop, mtDNA replication and transcription are being initiated and there is evidence that methylation can interfere with these processes (Sirard, 2019). In addition, a small decrease of *ND1* methylation was observed which led to an increase in *ND1* expression (Blanch et al., 2016). Thus, how mtDNA methylation affects mtDNA gene expression has not been fully elucidated yet.

Using long-read, single-molecule sequencing technologies like Nanopore sequencing (Deamer et al., 2016), it is possible to overcome limitations of the indirect measurement of methylation with bisulfite treatment, as it can be detected directly from squiggle signals (Xu and Seki, 2020). Nanopore technology enables the direct sequencing of full-length linearized mtDNA in contrast to the shorter fragments obtained from second-generation sequencing (De Coster and Van Broeckhoven, 2019). To our knowledge, there are currently only three studies that have used Nanopore sequencing to investigate mtDNA methylation (Aminuddin et al., 2020; Bicci et al., 2021; Goldsmith et al., 2021). Thus, detecting mtDNA methylation with this novel sequencing technology remains to be thoroughly investigated and validated.

While the main purpose of the study was to establish a pipeline for mtDNA methylation analysis using Nanopore sequencing technology, we included samples from PD patients with Parkin mutations to highlight potential areas of application in neuroscience. Our results suggest that mtDNA methylation exists and that it can be reliably detected by Nanopore sequencing. Moreover, the methodology holds promise for future studies exploring the molecular underpinnings of neurodegenerative diseases. Herein, we focus on the exploration of mtDNA CpG methylation using Nanopore. The present study aimed to (i) establish and validate a workflow to analyze mtDNA CpG methylation from native DNA and (ii) as a pilot, investigate mtDNA methylation from human blood-DNA as well as DNA from induced pluripotent stem cells (iPSC)-derived neurons in a specific Parkin-related PD model.

## Materials and Methods

### Sample Selection

All individuals included in this study provided informed consent and local ethics committee approval at the Research Ethics Boards of the Universities of Luebeck and Luxembourg were obtained. Eight individuals were included (L-13062, L-3244, L-3048, B-125, B-11, L-2131, L-2132 and L-2135). Five of these individuals are PD patients with *Parkin* mutations (Parkin-PD), with three being compound-heterozygous carriers (L-3244, L-13062 and B-11) and two homozygous carriers (L-3048 and B-125) (Supplementary Table 1). The other three are healthy control subjects without *Parkin* mutations (L-2131, L-2132 and L-2135). Movement disorder specialists performed the clinical assessments of patients. Genetic screening of the *Parkin* gene was performed with Sanger Sequencing and MLPA from blood-derived DNA, as previously described (Trinh et al., 2019). Blood-derived DNA from all individuals was included as well as DNA from iPSC-derived midbrain neurons generated from four patients (L-3244, L-3048, B-125 and B-11) and three controls (L-2131, L-2132 and L-2135).

### Generation of iPSC-derived Neurons

Fibroblast from four patients and three controls were utilized to generate iPSCs as previously described (Seibler et al., 2011). For culturing iPSCs, mTeSR^TM^ complete medium (StemCell Technologies) was used and, subsequently, cells were differentiated into small molecule neural precursor cells (smNPCs) by following an established protocol (Reinhardt et al., 2013). The smNPCs were cultured in N2/B27 medium—Neurobasal (Gibco)/DMEM-F12 (Gibco) 50:50 with 1% B27 lacking vitamin A (ThermoFisher), 1% 200 mM glutamine (Westburg), 1% penicillin-streptomycin (ThermoFisher) and 0.5% N2 (Life Technologies)—supplemented with 3 μM CHIR 99021 (Sigma), 150 μM ascorbic acid (AA) (Sigma) and 0.5 μM purmorphamine (PMA) (Sigma), which was replaced every other day. When smNPCs reached the 15th passage, the differentiation into midbrain neurons was induced. At ~75% confluence, smNPCs were detached by adding Accutase (Merck Millipore) and subsequently incubated for five min at 37°C and then pelleted by centrifuging for three min at 300 x g at room temperature. Via the Countess II FL Automated Cell Counter (ThermoFisher), smNPCs were counted and 750,000 cells were seeded in 6-well plates coated with Matrigel and covered with N2/B27 medium. N2/B27 medium with 200 μM AA, 1 μM PMA and 100 ng FGF8 (PeproTech) was used for eight days and then for two days N2/B27 medium with 200 μM AA and 0.5 μM PMA. Afterward, the cells were cultured 22 days in N2/B27 medium supplemented with 200 μM AA, 1 ng/mL TGF-β3 (Peprotech), 10 ng/mL GDNF (Peprotech), 20 ng/mL BDNF (PeproTech) and 500 μM dibutyryl-cAMP (Applichem). During this process, the medium was exchanged every other day.

### DNA Extraction

DNA extraction from blood was performed for all included individuals with the QIAGEN Blood DNA Mini Kit (Qiagen, Hilden, Germany) following the manufacturer's instructions. In addition, DNA was extracted from iPSC-derived neurons of four patients and three controls using the QIAAmp Minikit (Qiagen).

### Library Preparation and Nanopore Sequencing

#### Whole Genomes

DNA concentration of the blood- and neuron-derived DNA was quantified with Qubit fluorometric quantification using the dsDNA BR Assay Kit (Thermo Fisher Scientific, Waltham, MA, USA). Library preparation of 1.5 μg input DNA from the samples was performed with the Ligation Sequencing Kit (SQK-LSK109) (Oxford Nanopore Technologies, Oxford, United Kingdom) following a Nanopore whole-genome sequencing protocol (premium WGA). To conserve DNA methylation, whole-genome amplification with random primers was omitted from this established protocol (the intended first step of the premium WGA protocol).

#### Mitochondrial Genome Long-Range PCR

In order to create a negative control and a baseline for methylation calling, the mitochondrial genome of one patient (L-3244) was amplified with a long-range PCR. Two primer sets were used to generate two overlapping PCR products (MTL-F1 5′- AAA GCA CAT ACC AAG GCC AC−3′, MTL-F2 5′- TAT CCG CCA TCC CAT ACA TT−3′, MTL-R1 5′- TTG GCT CTC CTT GCA AAG TT−3′, MTL-R2 5′- AAT GTT GAG CCG TAG ATG CC−3′). The library was prepared using the Nanopore Ligation Sequencing Kit (SQK-LSK109) and multiplexed with the Native Barcoding Expansion (EXP-NBD104).

#### Synthetic DNA

To further validate the workflow, two types of commercially available 897 bp synthetic DNAs (D4505, Zymo Research, Irvine, CA, USA) were used where all cytosines were modified to 5-mC or unmodified. From these two synthetic DNAs, we created three different dilutions: (1) only methylated DNA (100% methylation), (2) 1:1 dilution of methylated and unmethylated DNA (50% methylation), and (3) only unmethylated DNA (0% methylation).

The DNA concentration was quantified as described above and 1 μg input DNA was used. The library was prepared using the Nanopore Ligation Sequencing Kit (SQK-LSK109) and multiplexed with the Native Barcoding Expansion (EXP-NBD104).

#### Sequencing

Nanopore sequencing was performed with the MinION or GidION using the R9.4.1 flow cells. The flow cell was primed and loaded according to the manufacturer's instructions. For base-calling, MinKNOW (version 20.10.6) with the integrated Guppy (version 4.2.3) was used. The Guppy base-calling software is available for Nanopore community members (https://community.nanoporetech.com). We aimed to obtain 3 Gigabases (Gb) of data before stopping the run which lasted ~24 h.

### Data Analysis

#### DNA Methylation Analysis

Base-called Nanopore reads were aligned to the reference sequence (i.e., mitochondrial genome, hg38 assembly), using Minimap2 (version 2.17). DNA methylation in a CpG dinucleotide context was called with Nanopolish (version 0.13.2), a software tool that uses a Hidden Markov Model (HMM) (Simpson et al., 2017). Nanopolish reports the log-likelihood ratio for each observed event (i.e., CpG site within a k-mer sequence). The methylation frequency (MF) was calculated with the default threshold of the log-likelihood ratio. Thus, with a log-likelihood ratio >2 the CpG site was classified as methylated and with a value < -2 the site was classified as unmethylated. In addition, the split-group parameter was used, which provides the MF of each detected CpG site in the reference sequence separately. The MF describes the proportion of reads that support methylation at the given CpG site. In addition, the Megalodon (version 2.2.9) software tool was used as well to investigate mtDNA CpG methylation with the basecall model res_dna_r941_min_modbases_5mC_v001 provided by Rerio (version 4.4.0).

To investigate the methylation of the synthetic DNA, alignment, methylation calling, and calculation of MF were performed. The D5405 reference sequence provided by the manufacturer was used. Reads that passed Guppy calling, as well as failed reads, were analyzed.

#### Adjusting the mtDNA Methylation Frequency

To adjust the detected methylation frequency of the blood- and neuron-derived samples, we calculated the false-positive rate (FPR = Number of called methylated cytosines/Number of all called cytosines) as recommended (Goldsmith et al., 2021) from the long-range PCR products. The resulting FPR of Nanopolish and Megalodon for blood- and neuron-derived DNA was subtracted from the methylation frequency of each site. In addition, all sites that showed a methylation frequency >0.2 in a PCR product were excluded from downstream analyses.

#### Plus- and Minus-Strand Methylation and Comparison With 45S rRNA

To explore heavy-strand (H-strand) and light-strand (L-strand) methylation, methylation calls from Nanopolish were stratified by plus- and minus-strand. The plus-strand represents the L-strand and the minus-strand the H-strand.

In order to compare the MF of mtDNA to a nuclear-encoded gene, we used the *45S rRNA* gene (13 kb core region of the 45S cluster 5). There are multiple copies of the *45S rRNA* gene in the human genome, comparable in size and coverage to the mtDNA. DNA methylation of the *45S rRNA* gene was stratified by plus- and minus-strand to allow comparison to the H- and L-strand of mtDNA.

#### Coverage and mtDNA Methylation Analysis

To investigate the relationship between coverage and MF, different subsets of the obtained sequencing data (i.e., Fast5 and FastQ files) with increasing coverage were used to detect methylation. The mean coverage and MF of each data set were calculated.

#### mtDNA Variants Detection

To additionally investigate the frequency of homoplasmic and heteroplasmic variants in the mtDNA, we processed the indexed alignments in BAM format with the Mutserve pipeline (version 2.0.0) (Weissensteiner et al., 2016). Due to the amplification-free approach of our study, the coverage of the mitochondrial genome in our study is below that typically reported in deep mitochondrial sequencing studies. To reduce the detection of artifacts, we set the minimum heteroplasmy level to 0.5 (i.e., 50%).

#### Statistical Analysis

For statistical analysis, GraphPad Prism software (version 9) was used. To compare median MF between plus- and minus-strand, blood- and neuron-derived mtDNA or patients and controls, the non-parametric Mann-Whitney U test for pairwise comparison was performed. Non-parametric Spearman correlation was used for correlation analyses. All *p*-values were exploratory and not corrected for multiple testing.

## Results

We first investigated mtDNA methylation in eight blood-derived samples to establish a workflow. In total, we obtained a mean of 8.6 Gb of data and a read length of 4.9 kb with whole-genome Nanopore sequencing. Alignment to the mitochondrial genome (hg38) resulted in a mean coverage of 250.3X (SD = ±80.5X, Supplementary Figure 1). To validate the workflow we assessed the false positive rate, sequencing depth, mitochondrial DNA (NUMTs) contamination as well as plus- and minus-strand differences. We then applied this workflow on a specific Parkinson's disease neuronal model as a pilot.

### Mitochondrial DNA CpG Methylation Detected With Nanopolish and Megalodon

**Figure 1:**
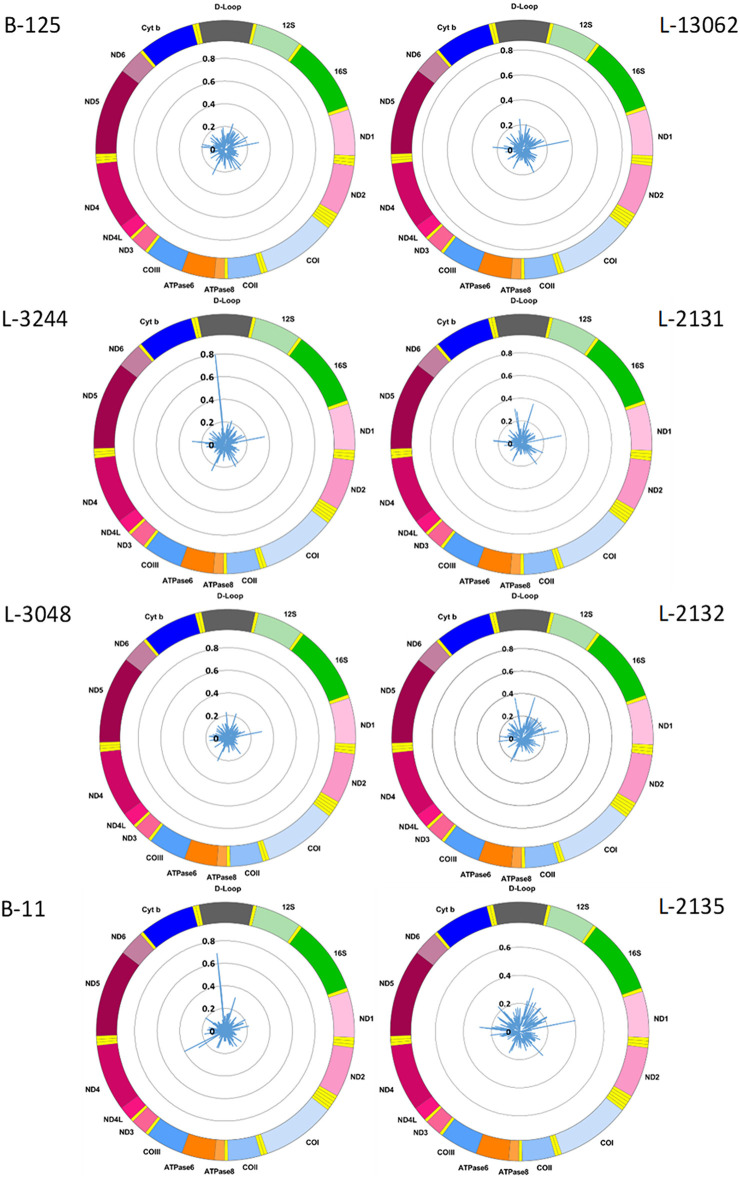
Mitochondrial CpG methylation frequency detected by Nanopore sequencing in eight individuals. Radar chart showing mitochondrial DNA methylation from eight blood-derived DNA samples detected by Nanopolish. Methylation frequency is indicated by blue spikes. 12S, small subunit rRNA; 16S, large subunit rRNA; ND1, NADH dehydrogenase, subunit 1; ND2, NADH dehydrogenase, subunit 2; COI, cytochrome c oxidase, subunit 1; COII, cytochrome c oxidase, subunit 2; ATPase8, ATP synthase, subunit 8; ATPase6, ATP synthase, subunit 6; COIII, cytochrome c oxidase, subunit 3; ND3, NADH dehydrogenase, subunit 3; ND4L, NADH dehydrogenase, subunit 4L; ND4, NADH dehydrogenase, subunit 4; ND5, NADH dehydrogenase, subunit 5; ND6, NADH dehydrogenase, subunit 6; Cyt b, cytochrome b; D-Loop, displacement loop.

We used the Nanopolish software tool to characterize CpG methylation. Overall, we detected low-level CpG methylation (mean MF ± SD = 0.057 ± 0.053) of mtDNA across the eight blood-derived samples (Figure 1). Additionally, we used Megalodon, which resulted in low-level mtDNA CpG methylation as well (mean MF ± SD = 0.086 ± 0.068). To access possible false-positive methylation calls, we adjusted the MF for the FPR calculated from the methylation calling of the long-range PCR product (FPR_Nanopolish_ = 0.029, FPR_Megalodon_ = 0.046) and excluded all CpG sites that showed an MF higher than 0.2 in the long-range PCR product from further analysis. After this correction, with Nanopolish there remained 11 unique CpG sites across the blood-derived samples with an MF >0.2 in the following genes: *12S rRNA* (chrM: 807, 1261, 1560), *16S rRNA* (chrM: 3008), *Cytochrome c oxidase, subunit 1* (chrM: 6052), *ATPase6/8* (chrM:8646), *NADH dehydrogenase, subunit 3* (chrM:10399), *NADH dehydrogenase, subunit 4* (chrM:12123), *tRNA-Glutamic acid* (chrM:14696), *tRNA-Threonine* (chrM:15929) and within the non-coding D-loop (chrM:16127). By contrast, with Megalodon there remained 28 unique CpG sites in the following mitochondrial genes: *12S rRNA* (chrM:807, 897, 1022, 1132, 1176, 1215, 1225, 1487), *16S rRNA* (chrM: 2565, 2569, 2571, 2842, 2914, 3077), *NADH dehydrogenase, subunit 1* (chrM: 3405), *tRNA-Methionine* (chrM:4425), *NADH dehydrogenase, subunit 2* (chrM: 4711), *Cytochrome c oxidase, subunit 1* (chrM: 6688), *NADH dehydrogenase, subunit 4* (chrM: 11715), *NADH dehydrogenase, subunit 5* (chrM: 12816, 13364), *tRNA-Glutamic acid* (chrM:14683), *Cytochrome b* (chrM: 15039, 15589, 15759), *tRNA-Threonine* (chrM:15929) and within the non-coding D-loop (chrM:16410). There was a weak correlation between the MF of individual CpG sites detected with Nanopolish and Megalodon (Spearman's *r* = 0.2924, Spearman's exploratory *p*-value < 0.0001, Supplementary Figure 2A) but a strong correlation between the mean MF of each sample (Spearman's *r* = 0.7904, Spearman's exploratory *p*-value = 0.0244, Supplementary Figure 2B). The calculated FPR was lower for Nanopolish analysis and the software required less computational resources in our set-up. After the formal comparison, we proceed with Nanopolish in our study. Next, we have analyzed the number of mtDNA variants and did not detect a difference in heteroplasmic variants likely due to our lower sequencing depth (Supplementary Table 2).

### Relationship Between the Coverage and Methylation Frequency

**Figure 2:**
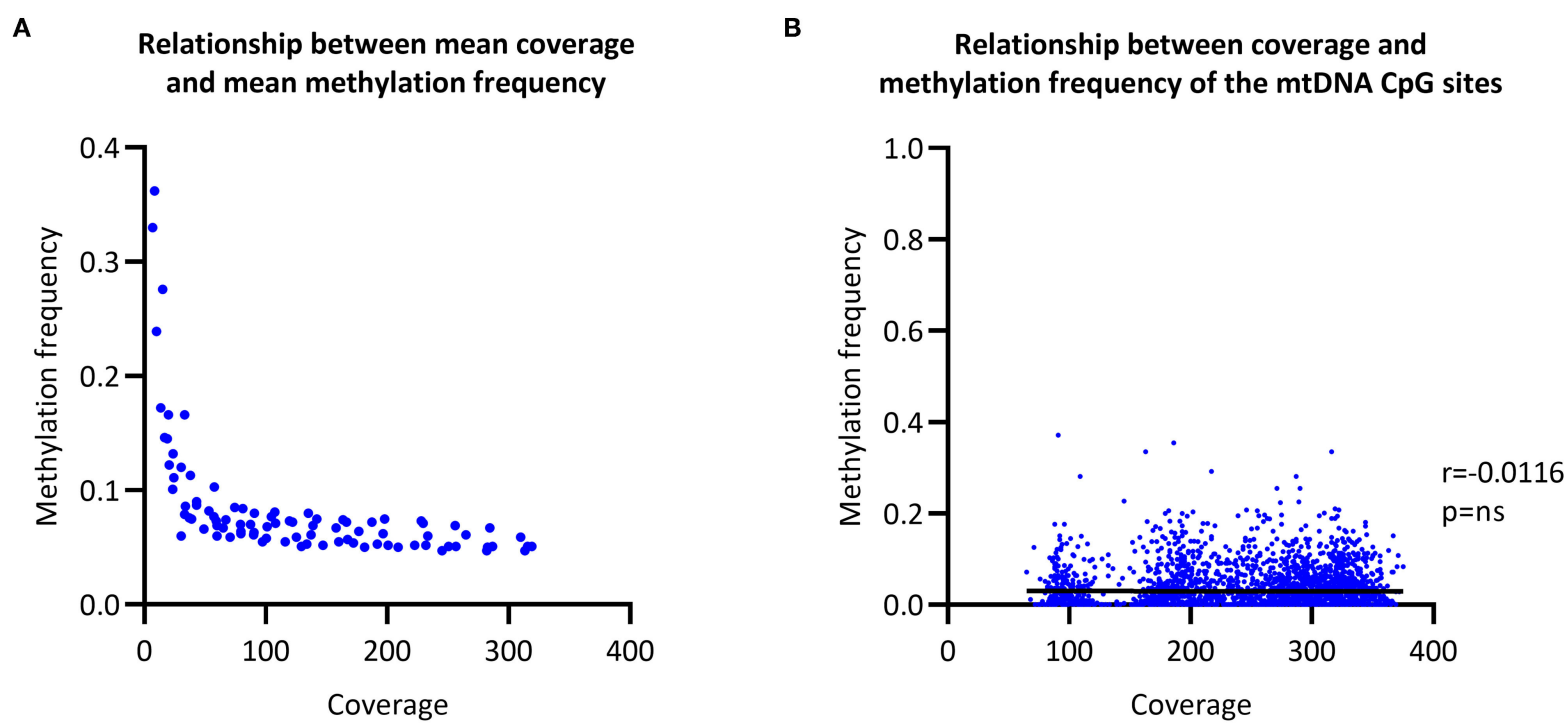
Relationship between coverage and methylation frequency. **(A)** Overall relationship between the mean coverage and mean methylation frequency of different subsections of the obtained sequencing data. **(B)** Overall relationship between the coverage of each individual CpG site and the corresponding methylation frequency. r, Spearman's rank correlation coefficient; p, Spearman's exploratory *p-*value.

To evaluate the relationship between coverage and MF, we increased the coverage of each sample in a step-wise fashion and then called CpG methylation. The raw mean MF for the eight individuals was at ~0.06 with >100X coverage (Figure 2A). In comparison, the raw mean MF detected from the long-range PCR products was at ~0.03 with >4000X coverage (Supplementary Figure 3). There was no correlation between coverage and detected MF when all of the sequencing data was used to call methylation (Spearman's *r* = 0.0116, Spearman's exploratory *p*-value > 0.05, Figure 2B).

### Nuclear Mitochondrial DNA

We explored possible NUMTs contamination in our data. NUMTs are transposition of mtDNA into the nuclear genome but most of these segments have sizes shorter than 500 bp (Dayama et al., 2014). Therefore, we filtered the reads by an alignment length to the mtDNA FASTA file of more than 1 kb to exclude possible NUMTs from the analysis. The detected MF after the filtering changed marginally and was strongly correlated to the MF without this filtering step (Spearman's *r* = 0.9445, Spearman's exploratory *p*-value < 0.0001, Supplementary Figure 4).

### Heavy- and Light-Strand Methylation Differences

**Figure 3:**
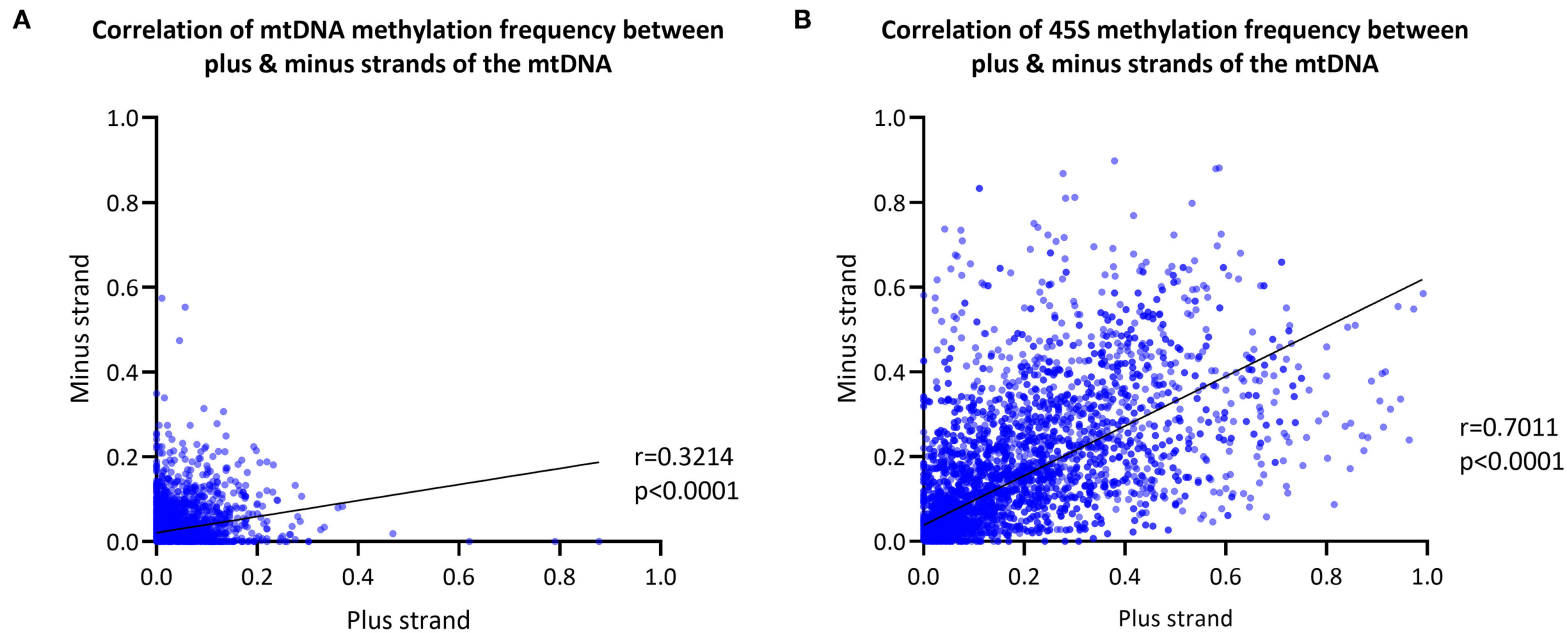
Mitochondrial and *45S rRNA* CpG methylation comparison by strand. **(A)** Correlation between the methylation frequency of the mitochondrial plus- and minus-strand (plus-strand = light-strand, minus-strand = heavy-strand). Data of the six blood-derived samples combined. **(B)** Correlation between the methylation frequency detected from the *45S rRNA* gene plus- and minus-strand. Data of the six blood-derived samples combined. r, Spearman's rank correlation coefficient; p, Spearman's exploratory *p*-value.

We next explored differences in methylation of the H- and L-strands. As the reference sequence of the mitochondrial genome represents the L-strand, the L-strand is reported as the plus-strand and the H-strand as the minus-strand. The CpG MF detected from the mtDNA plus- and minus-strands was corrected for the FPR, as well. We observed a positive correlation between the plus- and minus-strand MF detected with Nanopolish (Spearman's *r* = 0.3214, Spearman's exploratory *p*-value < 0.0001, Figure 3A).

For comparison, the *45S rRNA* gene was used as a nuclear-encoded comparison, since it has comparable size and coverage to the mtDNA (size = 13 kb, mean coverage = 623X). The overall correlation between plus-strand and minus-strand methylation was stronger in the *45S* rRNA gene compared to mtDNA (Spearman's *r* = 0.7011, Spearman's exploratory *p*-value < 0.0001, Figure 3B).

Our analysis showed that there was a trend for differences in the overall mean MF between mtDNA plus- and minus-strands in six individuals (Supplementary Figure 5A, Mann Whitney U-test *p* < 0.0382). However, the difference was not consistent in the same direction and effect sizes were small (difference in mean MF < 0.010). Similar to the mtDNA, the CpG sites in *45S rRNA* showed a trend for differences in the mean MF between plus- and minus-strands in five individuals (Supplementary Figure 5B, Mann Whitney U-test *p* < 0.0101, difference in mean MF <0.014). The number of plus- and minus-strand reads from the mtDNA and *45S rRNA* gene were comparable (Supplementary Figures 5C,D, Mann Whitney U-test *p* > 0.05).

### Synthetic DNA

To further validate the workflow and to also include a highly methylated sample, we performed Nanopore sequencing of an 897 bp synthetic methylated and unmethylated control DNA sample at different dilution ratios (0%, 50% and 100%). We obtained a mean of 14 Mb data and a read length of 963 bp. As the three samples were multiplexed for sequencing, barcodes with a length of 40 bp were added on both sides of the DNA fragments, which led to the higher mean read length of 963 bp compared to the 897 bp reference sequence. Alignment resulted in a mean coverage of 5018X (SD = ±424X).

#### Synthetic DNA Methylation

**Figure 4:**
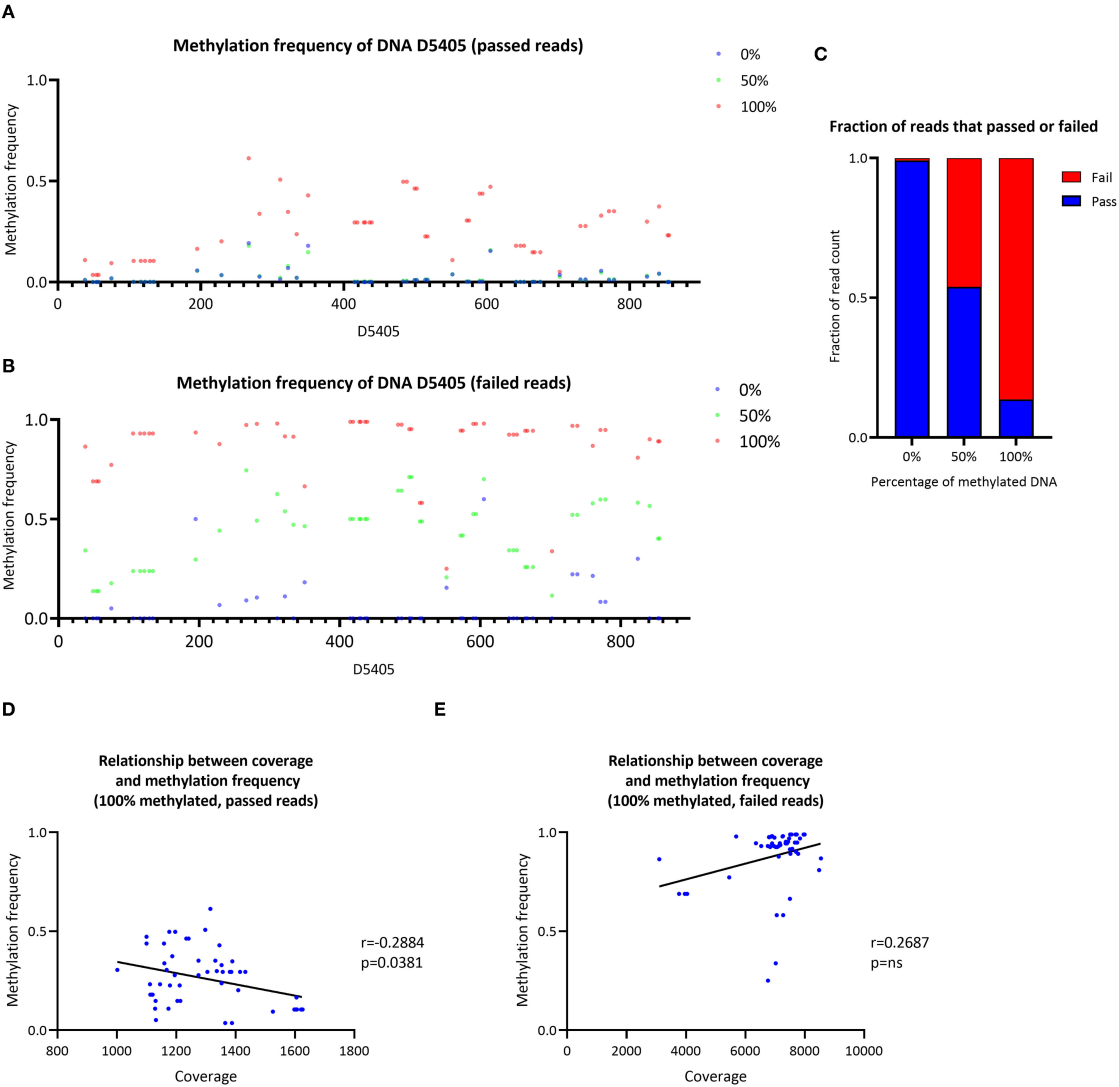
Analysis of synthetic DNA for the validation of the data analysis pipeline. **(A)** Methylation frequency detected by Nanopolish from three samples of synthetic DNA with different proportions of methylated DNA (0, 50 and 100%), only reads that passed Guppy quality threshold were included in the analysis. **(B)** Methylation frequency detected by Nanopolish from three samples of synthetic DNA with different proportions of methylated DNA (0, 50 and 100%), only reads that failed Guppy quality threshold were included in the analysis. **(C)** Stacked bar plot of the fraction of reads that passed (blue) or failed (red) the Guppy quality threshold, stratified by the ratio of methylated reads in the sample. **(D)** Relationship between the coverage and methylation frequency in the sample with 100% methylated reads, only reads that passed Guppy quality threshold were included in the analysis. **(E)** Relationship between the coverage and methylation frequency in the sample with 100% methylated reads, only reads that failed Guppy quality threshold were included in the analysis. r, Spearman's rank correlation coefficient; p, Spearman's exploratory *p*-value.

The CpG methylation of three samples with a ratio of 0, 50 or 100% of methylated synthetic DNA molecules was investigated (Figure 4A). In the 0% methylated sample, a mean *MF* = 0.021 (SD ± 0.042) was detected similar to the 50% methylated sample (mean MF ± SD = 0.022 ± 0.038). At particular CpG sites (e.g., D4505:195–350), the detected methylation level in the 0% methylated sample was higher than zero (range of *MF* = 0.014–0.193). In the 100% methylated sample, the detected methylation was higher (mean MF ± SD = 0.262 ± 0.141) compared to the 0% and 50% methylated samples. Nevertheless, the detected MF was lower than the corresponding ratio of methylated DNA molecules in the 50% and 100% methylated samples when we used Nanopolish to call the methylation. Similarly, when we used Megalodon, the detected MF was lower than expected in the 50% (mean MF ± SD = 0.040 ± 0.024) and 100% (mean MF ± SD = 0.535 ± 0.151) methylated sample as well (Supplementary Figure 6A).

#### Investigation of Guppy-Failed Reads

To investigate the possibility of preferential sequencing of unmethylated reads and methylated reads, which do not pass the quality threshold during base-calling, we analyzed Guppy-failed reads. During the Guppy base-calling, the software reports Phred quality scores (qscores) of each read, which indicates the accuracy of the base-calling. Reads with low qscores are classified as failed. Exploring these failed reads only, using Nanopolish, we detected low methylation in the 0% methylated sample (mean MF ± SD = 0.057 ± 0.123) and higher levels in the 50% methylated sample (mean MF ± SD = 0.430 ± 0.168) and the 100% methylated sample (mean MF ± SD = 0.880 ± 0.156). Thus, the detected methylation was overall higher in Guppy-failed reads compared to Guppy-passed reads in all samples (Figure 4B). Similarly, when we used Megalodon, the detected MF was higher in the 0% (mean MF ± SD = 0.054 ± 0.070), 50% (mean MF ± SD = 0.645 ± 0.168) and 100% methylated sample (mean MF ± SD = 0.687 ± 0.182) (Supplementary Figure 6B).

Including passed and failed reads, the proportion of failed reads was *n* = 55/6,550 in the 0% methylated sample, *n* = 26,068/56,577 in the 50% methylated sample and *n* = 44,505/51,555 in the 100% methylated sample (Figure 4C). Thus, the majority of methylated reads will not be analyzed under default Guppy parameters (cutoff value for the mean qscore = 7). In addition, there was a negative correlation between coverage of CpG sites and detected MF with Nanopolish from passed reads in the 100% methylated sample (Spearman's *r* = −0.2884, Spearman's exploratory *p*-value = 0.0381, Figure 4D) and in contrast, there was a trend toward a positive association when the MF was detected from the failed reads (Spearman's *r* = 0.2687, Spearman's exploratory *p*-value > 0.05, Figure 4E). When we used Megalodon, we observed the same trend for a negative association between coverage and MF from passed reads (Spearman's *r* = −0.1092, Spearman's exploratory *p*-value > 0.05, Supplementary Figure 5D) compared to a positive association in failed reads (Spearman's *r* = 0.1696, Spearman's exploratory *p*-value > 0.05, Supplementary Figure 5E).

### Re-analysis of Mitochondrial Methylation Including Guppy-Failed Reads

To consider the results obtained from the investigation of the synthetic DNA samples, mtDNA methylation was re-analyzed, including Guppy-passed and -failed reads. We obtained a mean coverage of 263X (SD = ±79X) of the mitochondrial genome, which was on average 13X more coverage compared to just including the passed reads. The overall change in the MF was marginal as the correlation was very strong between the MF detected from the passed reads only and the passed and failed reads together (Spearman's *r* = 0.9934, Spearman's exploratory *p*-value < 0.0001, Supplementary Figure 7).

### Investigation of Neuron-Derived Mitochondrial DNA

**Figure 5:**
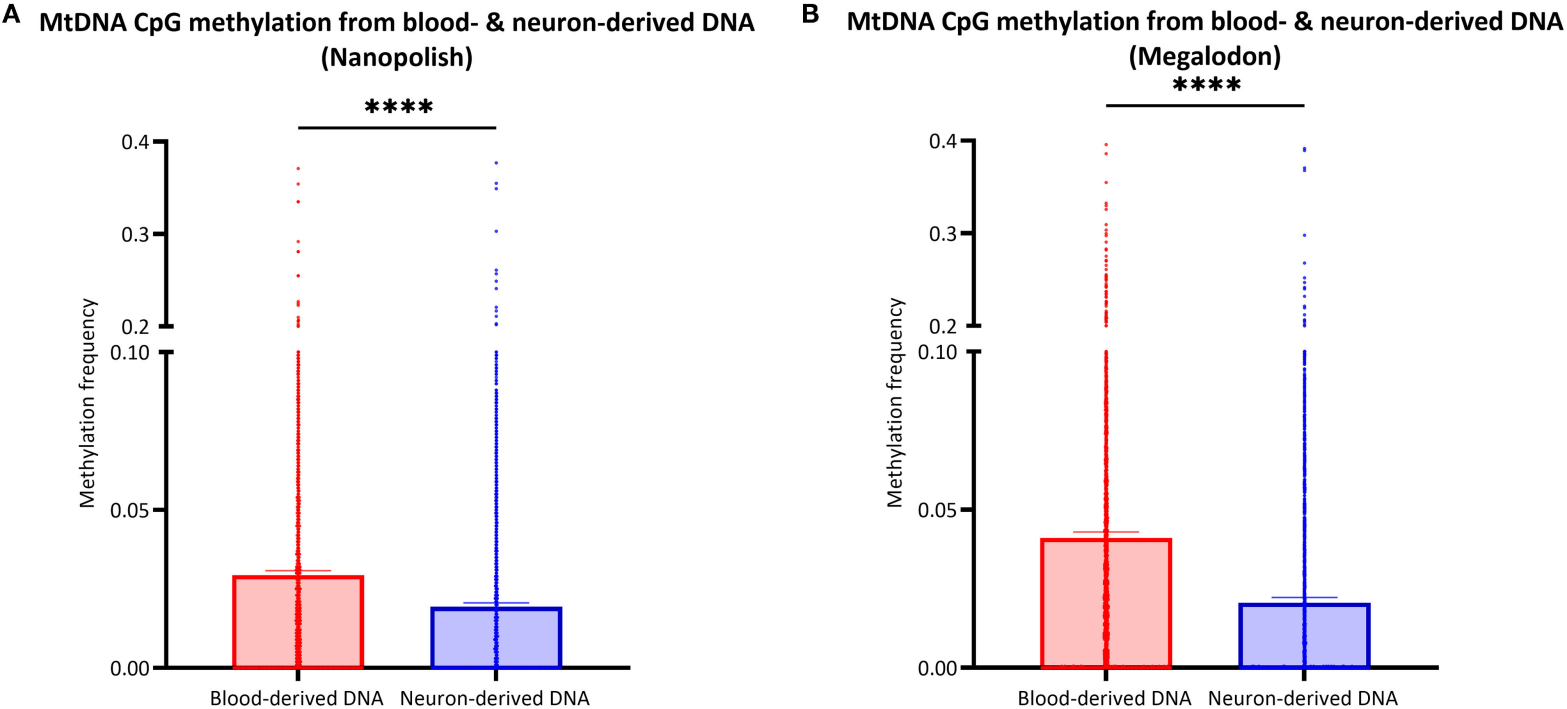
Comparison of mtDNA CpG methylation between blood- and neuron-derived DNA. **(A,B)** The bar plot shows the mtDNA CpG methylation from blood- or neuron-derived DNA (iPSC-derived midbrain neurons) which was either detected with Nanopolish **(A)** or Megalodon **(B)**. Bars indicate means and 95% confidence interval. The asterisks represent the level of significance (*p ≤ 0.05, **p ≤ 0.01, ***p ≤ 0.001, ****p ≤ 0.0001), *p*-value = Mann Whitney *U*-test performed for pairwise comparisons, blood-derived DNA is indicated in red and neuron-derived DNA in blue.

After the validation of our workflow, we compared mtDNA CpG methylation of blood- and neuron-derived DNA. Analogous to the blood-derived mtDNA, we obtained a mean of 7.1 GB of base-called data per sample and a mean read length of 5.6 kb (SD = ±2.1 kb). However, we obtained higher mean coverage of the neuron-derived mtDNA per sample with 830X (*SD* = ±465X). After the data were corrected for FPR, mtDNA methylation frequency detected with Nanopolish was significantly lower in neuron-derived DNA (mean MF ± SD = 0.019 ± 0.035) compared to blood-derived DNA (mean MF ± SD = 0.029 ± 0.041, Mann Whitney U-test p < 0.0001, Figure 5A). A significant difference between blood- and neuron-derived mtDNA CpG methylation was present and in the same direction with Megalodon as well (Figure 5B). Significantly higher methylation levels in blood-derived mtDNA were also observed when we included a second independent batch of neuron-derived DNA (Supplementary Figure 8A). We analyzed the relationship between blood- and neuron-derived mtDNA of each CpG site for individual participants and observed that the methylation frequency in blood- and neuron-derived mtDNA was correlated (Spearman's r > 0.5659, Spearman's exploratory *p*-value < 0.0001, Supplementary Figure 9).

### Comparison of Mitochondrial DNA Methylation in Patients With Parkinson's Disease and Healthy Controls

**Figure 6:**
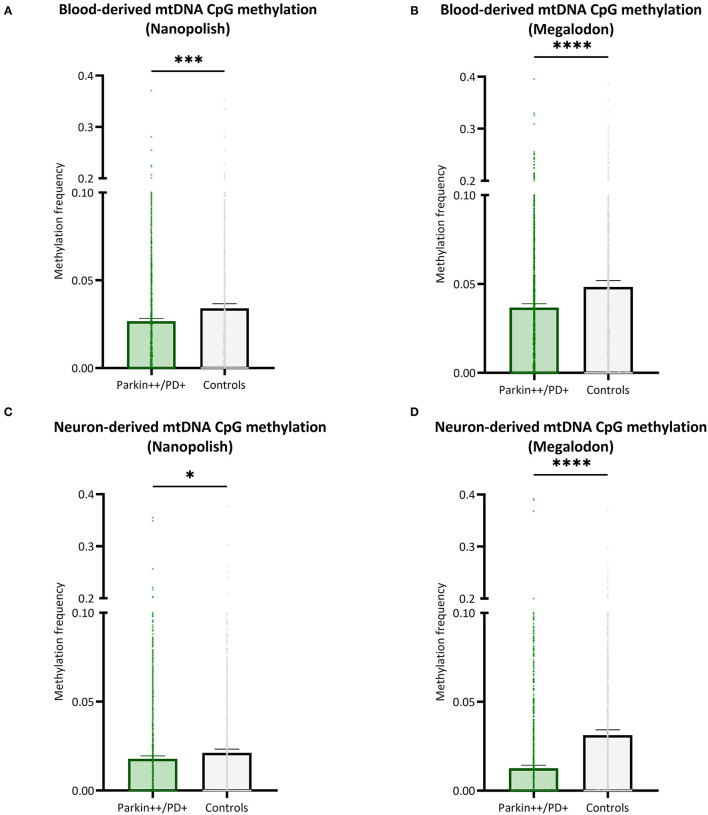
Comparison of mtDNA CpG methylation between patients with Parkin-PD and healthy controls. **(A,B)** The bar plot shows the mtDNA CpG methylation from blood-derived DNA in patients with Parkin-PD and healthy control subjects, which was either detected with Nanopolish **(A)** or Megalodon **(B)**. **(C,D)** The bar plot shows the mtDNA CpG methylation from neuron-derived DNA (iPSC-derived midbrain neurons) in patients with Parkin-PD and healthy control subjects, which was either detected with Nanopolish **(A)** or Megalodon **(B)**. Bars indicate means and 95% confidence interval. The asterisks represent the level of significance (*p ≤ 0.05, **p ≤ 0.01, ***p ≤ 0.001, ****p ≤ 0.0001), *p*-value = Mann Whitney *U-*test performed for pairwise comparisons, Parkin++ = Parkin biallelic; PD+ = PD patient, patients are indicated in green and control subjects in gray.

We included patients with Parkin-PD and healthy control subjects and compared the mtDNA CpG methylation between the groups in a global fashion. There were significantly lower methylation levels in Parkin-PD compared to healthy controls. The difference was present using either Nanopolish or Megalodon. The blood-derived mtDNA mean MF detected with Nanopolish in Parkin-PD was 0.027 (*SD* = 0.038) compared to 0.034 (*SD* = ±0.047) in healthy controls (Mann Whitney *U*-test *p* = 0.0002, Figure 6A) and with Megalodon the mean MF was 0.036 (*SD* = 0.051) in patients compared to 0.048 (*SD* = 0.065) in control subjects (Mann Whitney *U*-test *p* < 0.0001, Figure 6B). Additionally, in neuron-derived mtDNA, the MF detected with Nanopolish was significantly lower in patients (mean MF ± SD = 0.017 ± 0.034) compared to controls as well (mean MF ± SD = 0.021 ± 0.038, Mann Whitney *U*-test *p* = 0.0268, Figure 6C). Similarly, the MF detected with Megalodon was significantly lower in patients (mean MF ± SD = 0.013 ± 0.031) compared to controls (mean MF ± SD = 0.031 ± 0.053, Mann Whitney *U-*test *p* < 0.0001, Figure 6D). This significant difference was also present when we included a second independent batch of neuron-derived mtDNA (Supplementary Figure 8B). Lastly, we analyzed the relationship between the age at examination of the participants and the overall methylation level on the mtDNA. We did not observe a correlation between the age and the detected mean MF in blood- or neuron-derived DNA (*p* > 0.05, Supplementary Figure 10).

## Discussion

In this study, we (i) present and validate a workflow to assess mtDNA CpG methylation using whole-genome Nanopore sequencing and (ii) use the present workflow in a specific Parkin-PD model to compare mtDNA methylation of patients and controls as a pilot.

### Validation of mtDNA Methylation Analysis Workflow

We performed Nanopore sequencing of native whole-cell DNA and detected overall low-level mtDNA CpG methylation concordant with previous reports that used bisulfite sequencing (van der Wijst et al., 2017; Sun et al., 2018). The D-loop is the longest non-coding region of the mitochondrial genome and there is evidence that CpG methylation happens most prominently at sites within this region (Liu et al., 2016; van der Wijst et al., 2017). Interestingly, we detected higher methylation levels at two CpG sites within the D-loop (chrM: 16359 and 16410), however, these sites were excluded as false-positive calls. The FPR was lower when the methylation was called with Nanopolish compared to Megalodon and there were fewer CpG sites that showed an MF > 0.2 in the amplicon sequencing data, indicating Nanopolish is less prone to false-positive calls in the context of mtDNA CpG methylation. Although there are exceptions at particular sites after the correction of the MF, our results provide further evidence that mtDNA CpG methylation is low in general. Despite the weak concordance of the detected MF of each individual CpG site between Nanopolish and Megalodon, the concordance between the mean MF of each sample detected with the software tools was strong. The discrepancies in detected MF are likely due to different analysis algorithms used by both tools. Megalodon is based on a neural network, uses Guppy base calls of 5mC and maps the reads to a reference to provide per-site methylation data (https://github.com/nanoporetech/megalodon). Nanopolish is based on HMM to classify each CpG site of each read as methylated or unmethylated (Simpson et al., 2017). Additionally, the training strategy and the data sets themselves used to train these tools could contribute to the lower per-site concordance. A recent benchmark study investigated the strength and limitations of the widest used state-of-the-art tools to detect CpG methylation from Nanopore sequencing data, including Megalodon and Nanopolish (Yuen et al., 2021). The results from Megalodon were found to be more consistent with whole-genome bisulfite sequencing data compared to Nanopolish. Both tools showed, however, more disperse and lower per-site accuracy. Given the fact that, in our study, the overall methylation levels of samples are highly correlated between the tools, we used a global approach to assess differences in mtDNA CpG methylation between groups.

To validate if we obtained sufficient sequencing depth, we investigated the relationship between coverage and MF of mtDNA. As the mean MF plateaus at a coverage >100X, our results suggest that at least 100X coverage is necessary to detect mtDNA CpG methylation with Nanopore sequencing. With higher coverage, we did not observe an association between coverage and MF and therefore we concluded that we obtained sufficient sequencing depth to reliably detect mtDNA CpG methylation. Interestingly, a negative association between coverage and the detected MF was shown with whole-genome bisulfite sequencing (Mechta et al., 2017) and there were speculations that bisulfite conversion-resistant cytosines were the culprit. However, the detection of mtDNA methylation from the Nanopore sequencing data does not depend on bisulfite conversion and we observed a plateau of the mean MF after 100X coverage.

Next, we explored possible NUMTs contamination in our data. As these segments are usually shorter than 500 bp (Dayama et al., 2014), we excluded all reads that had an alignment length shorter than 1 kb to the mitochondrial genome reference file from the analysis and recalled CpG methylation. As the detected MF after the filtering only changed marginally, NUMTs contamination did not influence our results.

In the double-stranded mitochondrial genome, the H-strand has a higher purine content (G and A) compared to the L-strand and encodes 12 out of the 13 mitochondrial-encoded proteins (Berk and Clayton, 1974). We explored possible differences in mtDNA H- (minus-) and L- (plus-) strand CpG methylation in comparison to the nuclear-encoded *45S rRNA* gene. Although the correlation between plus- and minus-strand MF was higher in the *45S rRNA* gene, the trend for strand differences in the mtDNA mean MF were minor and without bias toward any specific strand. Still, a previous report suggested differences in H- and L-strand methylation mainly at non-CpG sites (Dou et al., 2019) which could be partially explained by the significantly lower frequency of cytosines in the H-strand (2,196) compared to the L-strand (5,181) (Dou et al., 2019). Methodologically, there was no preferential sequencing for either of the strands, as the number of plus- and minus-strand reads were comparable.

For further validation of our workflow, we used synthetic control DNA samples, which had 0, 50 or 100% methylation of DNA molecules. Highly methylated reads are prone to lower Guppy qscores, highlighted by the higher proportion of failed reads in the 100 and 50% methylated sample compared to the 0% methylated sample. As the detected MF in the 0% methylated sample was higher in the failed reads compared to the passed reads, there is the possibility of more false-positive calls though.

However, since mtDNA CpG methylation levels are low, the addition of the failed reads only marginally influenced the methylation. In fact, concerns about false positives increase when including failed reads, and therefore this is not recommended for quantification of mtDNA methylation. The inclusion of failed reads may be of importance when analyzing highly methylated samples of nuclear DNA in other research contexts.

### Mitochondrial CpG Methylation in Parkinson's Disease

With our analyses in PD patient-derived samples, we bring forth a case that mtDNA methylation does not only exist but can be reliably detected by Nanopore sequencing. This methodology is useful when investigating the molecular underpinnings of neurodegenerative diseases. Our proof-of-concept study in blood and neuronal samples from Parkin mutation carriers serves an example for better illustration. Although the overall methylation levels were low in the blood- and neuron-derived mtDNA, we observed significantly higher methylation in blood-derived mtDNA compared to neuron-derived mtDNA. On the one hand, there is evidence for a negative association between mtDNA methylation and gene expression (Sirard, 2019) and our results may indicate a higher expression of mtDNA genes in neuron-derived DNA. On the other hand, a previous study reported lower mtDNA CpG methylation levels in cell lines compared to tissue-derived DNA (Goldsmith et al., 2021). When exploring iPSC-derived midbrain neurons, lower CpG mtDNA methylation was detected compared to blood and perhaps the reprogramming process also modifies the epigenetics of mtDNA. Additionally, we have observed a correlation between the MF detected at the individual CpG sites in mtDNA derived from blood and neurons. Thus, mtDNA methylation, although lower in neuron-derived mtDNA, appear to be conserved across different cell types.

Importantly, we observed lower methylation levels in Parkin-PD compared to healthy controls, which is evidenced in blood- and neuron-derived mtDNA. A previous study that used targeted bisulfite pyrosequencing to investigate mtDNA CpG methylation has reported lower methylation in patients with PD as well, and more specifically within the D-Loop (Blanch et al., 2016). A recent publication using whole-genome bisulfite sequencing did not show an association between mtDNA methylation and idiopathic PD (Guitton et al., 2021) but an association between mtDNA methylation and transcript levels. In our study, all patients included were biallelic *Parkin* mutation carriers. As Parkin is involved in the degradation of damaged mitochondria and mtDNA maintenance, patients carrying Parkin mutations could be prone to mtDNA dyshomeostasis. Altered methylation levels in Parkin-PD patients could affect the regulation of mtDNA gene expression and thereby contribute to mitochondrial dysfunction. By contrast, further functional studies will be required to investigate if mtDNA methylation and gene expression are causally linked.

As nuclear genome methylation is associated with age (Benayoun et al., 2015; Jiang and Guo, 2020) and similar findings have been reported for mtDNA (Mawlood et al., 2016), we analyzed the relationship between age and mtDNA CpG methylation in both, the blood and neuronal samples, but did not observe a correlation. However, the sample size in our study was limited and we assessed the overall mean methylation level rather than site-specific methylation frequencies.

### Review of Literature on mtDNA Nanopore Sequencing

We performed a literature search for the terms “mtDNA” and “Nanopore” and found 47 publications, eight of which performed Nanopore sequencing of human mtDNA (Lindberg et al., 2016; Carter and Hussain, 2017; Zascavage et al., 2019; Aminuddin et al., 2020; Bi et al., 2020; Georgieva et al., 2020; Bicci et al., 2021; Goldsmith et al., 2021) (Supplementary Figure 11). In these studies, the coverage of the mitochondrial genome ranged from 60.4X (Aminuddin et al., 2020) to over 10000X (Goldsmith et al., 2021) (Supplementary Table 3). In the latter case, extraction of mtDNA from a subcellular fraction was performed before Nanopore sequencing, which enhanced the fraction of sequenced reads that mapped to the mitochondrial genome.

Three out of the six publications investigated mtDNA methylation in the context of cancer research (Aminuddin et al., 2020; Bicci et al., 2021; Goldsmith et al., 2021). In our study, we included patients with Parkin-PD and investigated global differences in mtDNA CpG methylations between patients and control subjects. For the published studies in cancer cells, Nanopolish was used to call methylation from the Nanopore sequencing data (Aminuddin et al., 2020; Bicci et al., 2021; Goldsmith et al., 2021). In addition to Nanopolish, one study used Guppy in combination with Medaka (Goldsmith et al., 2021). Low-level mtDNA methylation in these three studies has been described (Aminuddin et al., 2020; Bicci et al., 2021; Goldsmith et al., 2021), albeit one study evaluated only three specific sites. We also detected overall low-level mtDNA methylation in blood-derived and neuron-derived DNA, in concordance with the reported literature (Aminuddin et al., 2020; Bicci et al., 2021; Goldsmith et al., 2021).

### Strengths and Limitations

In our study, we present an optimized protocol for the investigation of mtDNA CpG methylation. We obtained high coverage of the mtDNA as well as the nuclear-encoded *45S rRNA* gene and the synthetic DNA samples. The mean coverage of the mtDNA ranged from 177X to 320X across individuals. Linearization of mtDNA has been recommended to counteract the influence of secondary and tertiary structure in bisulfite-dependent methods (Liu et al., 2016; Mechta et al., 2017; Owa et al., 2018). Thus, we performed T7 Endonuclease I digestion of whole-cell DNA for single-molecule sequencing. Still, the sample size of our study was small with eight included individuals and our analysis was limited to DNA methylation at CpG sites. As previous studies have shown that there is non-CpG methylation present in the mtDNA (Dou et al., 2019; Patil et al., 2019), future workflows should be expanded to include non-CpG methylation as well. In the current study, mtDNA methylation was analyzed in DNA extracted from whole blood, which is the most common source of DNA in genetic research. Unfortunately, some samples were frozen EDTA vials, which precluded cell compositing analyses. By contrast, future studies should be based on fresh blood draws to allow for a correlation of cell type and mtDNA methylation status in a given sample. Another limitation of this study is that Parkin mutation carriers are rare and an assessment of a larger Parkin-PD cohort is therefore only possible within the framework of international collaborations. Furthermore, due to the high cell culture costs, time and labor-intensive procedure, such mechanistic studies in iPSC-derived cell models are typically limited to a small number of samples. However, while we currently do not have access to additional iPSC lines with biallelic Parkin mutations, we have included an additional independent differentiation of all lines in the study. In this fashion, we could explore the impact of inter-experimental variability on our data set. This validation showed methylation profiles in the control and Parkin-PD patients comparable to the original analysis. Given these limitations, we investigated two different types of genetic material in addition to the 16.5 kb mitochondrial genome to validate our workflow, i.e. an 897 bp synthetic DNA sample and the native 13 kb *45S rRNA* nuclear-encoded gene. To have a comparison of two software tools, we have used Nanopolish and Megalodon to call methylation, which showed high concordance between the mean MF detected for each sample. Additionally, we have corrected our data for false-positive methylation calls individually for Nanopolish and Megalodon. Lastly, all experiments and analyses were performed at the same institute to reduce batch effects. These results highlight that using Nanopore sequencing overcomes the bias that might be introduced by bisulfite-based methods due to conversion-resistant cytosines of the mtDNA. But it is important to note that also with Nanopore sequencing we observed false-positive methylation calls as illustrated by the FPR calculated from the long-range PCR sequencing data. Thus, the software predicting the MF tools might need to be trained with more mtDNA-specific data sets to enhance the accuracy even more.

## Conclusion

In conclusion, our results suggest the applicability of Nanopore sequencing for the investigation of mtDNA methylation. Overall, we detected low-level CpG methylation of mtDNA with exceptions for certain sites. Our results suggest that highly methylated DNA molecules were more likely to fail Guppy base-calling and therefore bioinformatic pipelines that take Guppy-failed reads into account are recommended for highly methylated samples. Despite our small sample size, we observed significant differences in patients with Parkin-PD, which highlights the importance to further investigate the underlying molecular mechanism and its implication in neurodegeneration. Additionally, an expansion of the workflow to include non-CpG methylation analysis is warranted.

## Data Availability Statement

The datasets presented in this study can be found in online repositories. The names of the repository/repositories and accession number(s) can be found below: NCBI SRA, accession numbers: SAMN20956590-SAMN20956606.

## Ethics Statement

The studies involving human participants were reviewed and approved by Research Ethics Boards of the Universities of Luebeck and the Research Ethics Boards of the Universities of Luxembourg. Written informed consent to participate in this study was provided by the participants' legal guardian/next of kin.

## Author Contributions

JT, CK, AG, LS, and SP contributed to conception and design of the study. TL, KW, SS, RT, JL, and JT contributed to the acquisition and analysis of data. TL, CK, SP, JL, LS, AG, and JT contributed to interpretation of data. TL and JT wrote the first draft of the manuscript. All authors participated in revising the manuscript for intellectual content and approved its final version.

## Funding

Funding has been obtained from the German Research Foundation (ProtectMove; FOR 2488, GR 3731/5-1; SE 2608/2-1; KO 2250/7-1), the Luxembourg National Research Fund within the ATTRACT (Model-IPD, FNR9631103) and INTER programs (ProtectMove, INTER/DFG/19/14429377), the Personalized Medicine Consortium (PMC) of Luxembourg (PUMP-PRIME grant), the European Community (SysMedPD), the Canadian Institutes of Health Research (CIHR), Peter and Traudl Engelhorn Foundation, and the Joachim Herz Stiftung.

## Conflict of Interest

The authors declare that the research was conducted in the absence of any commercial or financial relationships that could be construed as a potential conflict of interest.

## Publisher's Note

All claims expressed in this article are solely those of the authors and do not necessarily represent those of their affiliated organizations, or those of the publisher, the editors and the reviewers. Any product that may be evaluated in this article, or claim that may be made by its manufacturer, is not guaranteed or endorsed by the publisher.
