# Supplementary material for: Nanopore Single-Molecule Sequencing for Mitochondrial DNA Methylation Analysis: Investigating Parkin-Associated Parkinsonism as a Proof of Concept
Source: Front Aging Neurosci. 2021 Sep 28;13:713084. doi: 10.3389/fnagi.2021.713084 (PMC8506010; doi:10.3389/fnagi.2021.713084)
Supplement: Supplementary file 1 [file Data_Sheet_1.docx]

Supplementary Material

# Supplementary Figures


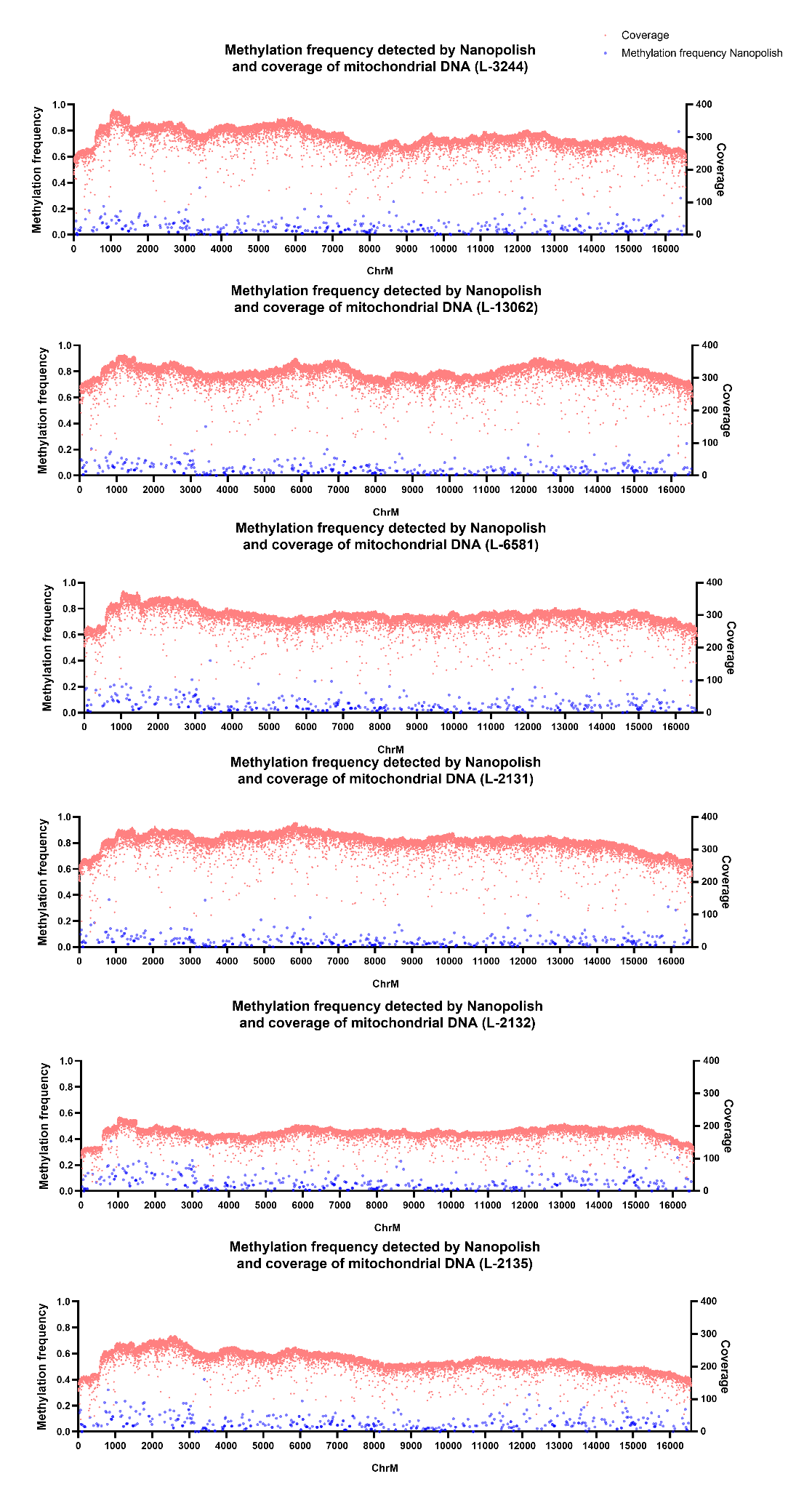

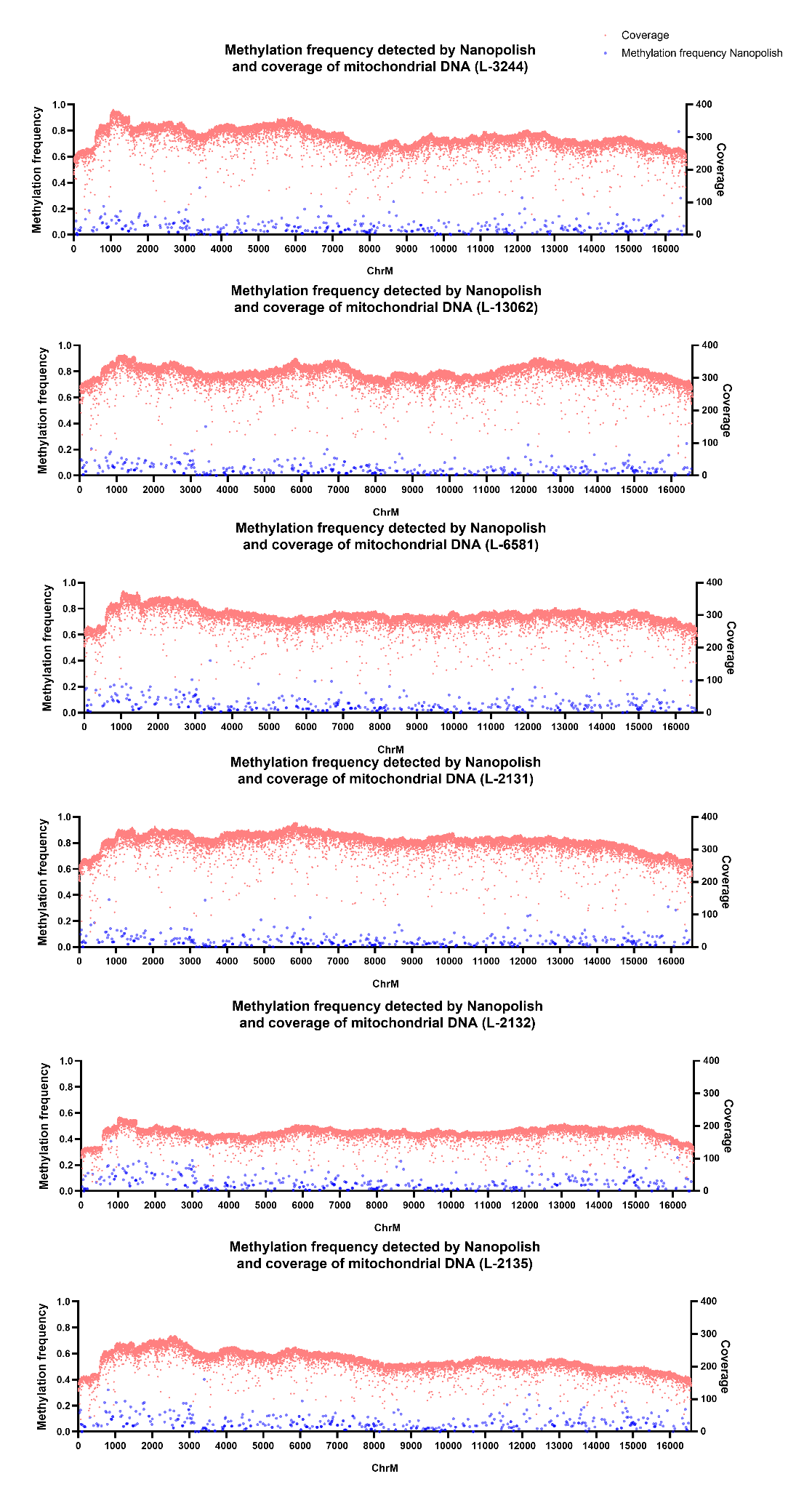


**Supplementary Figure 1. Mitochondrial CpG methylation frequency and coverage detected by Nanopore sequencing in five exemplary individuals**. The methylation frequency of the mitochondrial DNA from five blood-derived DNA samples, detected by Nanopolish. Methylation frequency is indicated by blue dots and coverage by red dots and the x-axis indicates the positions in the mitochondrial genome (hg38).

#

**Supplementary Figure 2. Concordance of mtDNA CpG methylation detected with Nanopolish and Megalodon.** A) Relationship between the methylation frequency detected with Nanopolish and Megalodon of each individual CpG site. B) Relationship between the mean methylation frequency of each sample detected by Nanopolish and Megalodon.

r=Spearman’s rank correlation coefficient, p=Spearman’s exploratory p-value

**Supplementary Figure 3. Relationship between coverage and methylation frequency of the long-range PCR amplicons**. A) Overall relationship between the mean coverage and mean methylation frequency of different subsections of the obtained sequencing data.

**Supplementary Figure 4. Concordance of mtDNA CpG with and without filtering for nuclear mitochondrial DNA contamination.** Relationship between the methylation frequency detected with Nanopolish from all reads or from reads with a minimum of 1kb of alignment length to the mtDNA reference.

r=Spearman’s rank correlation coefficient, p=Spearman’s exploratory p-value

#

**Supplementary Figure 5. Exploration of DNA CpG methylation stratified by strand.** A/B) Bar plot of the methylation frequency stratified by strands from the mtDNA or the 45S rRNA gene. Bars indicating mean and 95% confidence interval, p-value=Mann Whitney U-test performed for pairwise comparisons. The asterisks represent the level of significance (*: p≤.05, **: p≤.01, ***: p≤.001, ****: p≤.0001), p-value=Mann Whitney U-test performed for pairwise comparison C/D) Bar plot showing number of reads mapped to the mitochondrial genome or the 45S rRNA gene, stratified by strands.

**Supplementary Figure 3. Exploration of DNA CpG methylation stratified by strand.** A/B) Scatter plot of the methylation frequency stratified by strands from the mtDNA or the 45S rRNA gene . Bars indicating median and inter quartile range (IQR), p-value=Mann Whitney U-test performed for pairwise comparisons. C/D) Bar plot showing number of reads mapped to the mitochondrial genome or the 45S rRNA gene and evaluated by Nanopolish, stratified by strands.

**Supplementary Figure 6. Analysis of synthetic DNA for the validation of the data analysis pipeline.** A) Methylation frequency detected by Megalodon from three samples of synthetic DNA with different proportions of methylated DNA (0%, 50% and 100%), only reads that passed Guppy quality-threshold were included in the analysis. B) Methylation frequency detected by Megalodon from three samples of synthetic DNA with different proportions of methylated DNA (0%, 50% and 100%), only reads that failed Guppy quality-threshold were included in the analysis. C) Stacked bar plot of the fraction of reads that passed (blue) or failed (red) the Guppy quality-threshold, stratified by the proportion of methylated reads in the sample. D) Relationship between the coverage and methylation frequency in the sample with 100% methylated reads, only reads that passed Guppy quality-threshold were included in the analysis. E) Relationship between the coverage and methylation frequency in the sample with 100% methylated reads, only reads that failed Guppy quality-threshold were included in the analysis.

r=Spearman’s rank correlation coefficient, p=Spearman’s exploratory p-value

**Supplementary Figure 7. Concordance of mtDNA CpG reanalysis with failed reads included.** Relationship between the methylation frequency detected with Nanopolish from passed reads only or from passed and reads.

r=Spearman’s rank correlation coefficient, p=Spearman’s exploratory p-value

**Supplementary Figure 8. Comparison of mtDNA CpG methylation between blood- and neuron-derived as well as patient- and control-derived DNA.** A) The bar plot shows the mtDNA CpG methylation from blood- or two independent batches of neuron-derived DNA (iPSC-derived midbrain neurons). B) The bar plot shows the mtDNA CpG methylation from two independent batches of neuron-derived DNA (iPSC-derived midbrain neurons) in patients with Parkin-PD and healthy control subjects. Bars indicate means and 95% confidence interval. The asterisks represent the level of significance (*: p≤.05, **: p≤.01, ***: p≤.001, ****: p≤.0001), p-value=Mann Whitney U-test performed for pairwise comparison

**Supplementary Figure 9. Relationship of the individual CpG methylation frequency in blood- and neuron-derived mtDNA.** The plots show the correlation between per-site methylation frequency of blood- and neuron derived mtDNA from A-D) patients with PD and E-G) control subjects.

**Supplementary Figure 10. Relationship between the age and the overall methylation level.** Correlation between the age at examination and the mean methylation frequency (MF) of the mitochondrial DNA derived from A) blood and B) neurons. The MF detected with Nanopolish is indicated in blue and Megalodon in red.

**Supplementary Figure 11. Flowchart summarizing literature search for articles on mitochondrial DNA and Nanopore sequencing.** The Search term was: “mitochondrial DNA Nanopore” and the date of the literature search was: May 6th, 2021.

# Supplementary Tables

**Supplementary Table 1.** Demographics of study participants

|  | L-3244 | L-13062 | B-11 | L-3048 | B-125 |
| --- | --- | --- | --- | --- | --- |
| AAE | 49 | 17 | 71 | 57 | 54 |
| AAO | 36 | 11 | 64 | 15 | 43 |
| Gender | Female | Female | Male | Male | Female |
| Parkin variant I  - Protein level identifier  - cDNA level identifier | p.Arg275Trp  c.823C>T | p.Met1?  c.2T>C | c.1072 delT het | c.924 C>T hom | c.1072 delT hom |
| Parkin variant II  - Deletion  - cDNA level identifier | exon 1 del  c.(?_-103-1)_(7+1_8-1)del | exon 11 del  c.(1167+1_1168-1)_(1285+1_1286-1)del​ | Ex7 het Del.+ | n.a. | n.a. |
|  | L-2131 | L-2132 | L-2135 |  |  |
| AAE | 57 | 35 | 39 |  |  |
| AAO | n.a. | n.a. | n.a. |  |  |
| Gender | Male | Female | Male |  |  |

n.a.=Not applicable, AAE=Age at examination, AAO=Age at onset, Parkin variant I/II=Description of Parkin variants of the compound heterozygous (L-3244, L-13062, B11) or heterozygote (L-6581, L-3048, B-125) mutation carriers with PD, del=Deletion, all mutations have been curated here: https://www.mdsgene.org/

**Supplementary Table 2.** Overview of detected mitochondrial variants

| ID | Pos | Ref | Variant | Variant frequency level |
| --- | --- | --- | --- | --- |
| B11 | 73 | A | G | 1 |
| B11 | 151 | C | T | 0.905 |
| B11 | 152 | T | C | 0.979 |
| B11 | 263 | A | G | 1 |
| B11 | 750 | A | G | 0.885 |
| B11 | 1189 | T | C | 0.791 |
| B11 | 1438 | A | G | 0.857 |
| B11 | 1658 | T | C | 0.714 |
| B11 | 1811 | A | G | 0.788 |
| B11 | 1819 | T | C | 0.809 |
| B11 | 2706 | A | G | 0.906 |
| B11 | 3107 | N | T | 1 |
| B11 | 3480 | A | G | 0.815 |
| B11 | 4769 | A | G | 0.949 |
| B11 | 6413 | T | C | 0.903 |
| B11 | 7028 | C | T | 0.949 |
| B11 | 8251 | G | A | 0.8 |
| B11 | 8860 | A | G | 0.882 |
| B11 | 9055 | G | A | 1 |
| B11 | 9698 | T | C | 0.875 |
| B11 | 10398 | A | G | 0.886 |
| B11 | 10478 | C | T | 0.976 |
| B11 | 10550 | A | G | 0.917 |
| B11 | 11299 | T | C | 0.974 |
| B11 | 11467 | A | G | 0.842 |
| B11 | 11719 | G | A | 1 |
| B11 | 12308 | A | G | 0.667 |
| B11 | 12372 | G | A | 0.957 |
| B11 | 12528 | G | A | 0.462 |
| B11 | 12810 | A | G | 0.889 |
| B11 | 13643 | G | A | 0.667 |
| B11 | 14142 | C | G | 0.938 |
| B11 | 14167 | C | T | 0.981 |
| B11 | 14766 | C | T | 0.932 |
| B11 | 14798 | T | C | 0.844 |
| B11 | 15326 | A | G | 0.92 |
| B11 | 16093 | T | C | 0.94 |
| B11 | 16189 | T | A | 0.898 |
| B11 | 16224 | T | C | 0.923 |
| B11 | 16274 | G | A | 0.948 |
| B11 | 16311 | T | C | 1 |
| B11 | 16362 | T | C | 1 |
| B11 | 16519 | T | C | 1 |
| L13062 | 73 | A | G | 0.981 |
| L13062 | 153 | A | G | 0.983 |
| L13062 | 195 | T | C | 0.99 |
| L13062 | 263 | A | G | 1 |
| L13062 | 709 | G | A | 0.955 |
| L13062 | 750 | A | G | 0.841 |
| L13062 | 1438 | A | G | 0.797 |
| L13062 | 1719 | G | A | 1 |
| L13062 | 2706 | A | G | 0.809 |
| L13062 | 2712 | G | A | 0.832 |
| L13062 | 2778 | T | C | 0.892 |
| L13062 | 3107 | N | T | 0.875 |
| L13062 | 4052 | C | T | 0.882 |
| L13062 | 4427 | G | A | 0.562 |
| L13062 | 4769 | A | G | 0.899 |
| L13062 | 4823 | T | C | 0.944 |
| L13062 | 5237 | G | A | 0.923 |
| L13062 | 5238 | C | T | 0.522 |
| L13062 | 5984 | A | G | 0.867 |
| L13062 | 6221 | T | C | 0.827 |
| L13062 | 6371 | C | T | 0.929 |
| L13062 | 7028 | C | T | 0.861 |
| L13062 | 7337 | G | A | 0.77 |
| L13062 | 8860 | A | G | 0.945 |
| L13062 | 10338 | T | C | 0.938 |
| L13062 | 10427 | G | A | 0.936 |
| L13062 | 10954 | C | T | 0.95 |
| L13062 | 11719 | G | A | 0.993 |
| L13062 | 12084 | C | T | 0.845 |
| L13062 | 12705 | C | T | 0.98 |
| L13062 | 13966 | A | G | 0.75 |
| L13062 | 14470 | T | C | 0.919 |
| L13062 | 14766 | C | T | 0.914 |
| L13062 | 15310 | T | C | 0.96 |
| L13062 | 15326 | A | G | 0.931 |
| L13062 | 15721 | T | C | 0.946 |
| L13062 | 16183 | A | C | 0.737 |
| L13062 | 16223 | C | T | 0.974 |
| L13062 | 16265 | A | G | 0.859 |
| L13062 | 16278 | C | T | 0.951 |
| L13062 | 16519 | T | C | 1 |
| L3048 | 73 | A | G | 0.984 |
| L3048 | 263 | A | G | 1 |
| L3048 | 750 | A | G | 0.841 |
| L3048 | 1438 | A | G | 0.878 |
| L3048 | 1658 | T | C | 0.743 |
| L3048 | 1888 | G | A | 0.873 |
| L3048 | 2831 | G | T | 0.859 |
| L3048 | 3107 | N | T | 0.833 |
| L3048 | 3315 | G | A | 0.868 |
| L3048 | 4769 | A | G | 0.943 |
| L3048 | 6776 | T | C | 0.867 |
| L3048 | 8860 | A | G | 0.955 |
| L3048 | 8911 | T | C | 0.959 |
| L3048 | 9752 | C | T | 0.615 |
| L3048 | 11377 | G | A | 0.891 |
| L3048 | 11852 | G | A | 0.522 |
| L3048 | 13209 | C | T | 0.883 |
| L3048 | 15326 | A | G | 0.951 |
| L3048 | 16298 | T | C | 0.981 |
| L3048 | 16352 | T | C | 0.925 |
| L3048 | 16519 | T | C | 0.992 |
| B125 | 150 | C | T | 0.97 |
| B125 | 263 | A | G | 0.958 |
| B125 | 750 | A | G | 0.82 |
| B125 | 1438 | A | G | 0.77 |
| B125 | 1598 | G | A | 0.817 |
| B125 | 1986 | A | T | 0.877 |
| B125 | 2573 | G | A | 0.876 |
| B125 | 3107 | N | T | 0.938 |
| B125 | 3248 | G | A | 0.848 |
| B125 | 3316 | G | A | 0.811 |
| B125 | 4769 | A | G | 0.917 |
| B125 | 5237 | G | A | 0.545 |
| B125 | 5568 | A | G | 0.757 |
| B125 | 5703 | G | A | 0.484 |
| B125 | 8206 | G | A | 0.964 |
| B125 | 8860 | A | G | 0.939 |
| B125 | 11377 | G | A | 0.879 |
| B125 | 11719 | G | A | 0.851 |
| B125 | 11852 | G | A | 0.5 |
| B125 | 12112 | C | T | 0.379 |
| B125 | 12528 | G | A | 0.574 |
| B125 | 14318 | T | C | 0.897 |
| B125 | 14655 | G | A | 0.935 |
| B125 | 14872 | C | T | 0.843 |
| B125 | 15326 | A | G | 0.943 |
| B125 | 15773 | G | A | 0.923 |
| L2131 | 73 | A | G | 0.989 |
| L2131 | 263 | A | G | 1 |
| L2131 | 709 | G | A | 0.956 |
| L2131 | 750 | A | G | 0.853 |
| L2131 | 930 | G | A | 0.941 |
| L2131 | 1438 | A | G | 0.83 |
| L2131 | 1888 | G | A | 0.963 |
| L2131 | 1906 | G | A | 0.673 |
| L2131 | 2706 | A | G | 0.824 |
| L2131 | 3107 | N | T | 0.944 |
| L2131 | 3315 | G | A | 0.864 |
| L2131 | 3666 | G | A | 0.869 |
| L2131 | 3921 | C | A | 0.727 |
| L2131 | 4216 | T | C | 0.855 |
| L2131 | 4769 | A | G | 0.883 |
| L2131 | 4917 | A | G | 0.862 |
| L2131 | 5147 | G | A | 1 |
| L2131 | 5237 | G | A | 0.667 |
| L2131 | 5540 | G | A | 0.933 |
| L2131 | 5703 | G | A | 0.638 |
| L2131 | 5811 | A | G | 0.785 |
| L2131 | 7028 | C | T | 0.909 |
| L2131 | 7642 | G | A | 0.796 |
| L2131 | 8697 | G | A | 0.976 |
| L2131 | 8860 | A | G | 0.903 |
| L2131 | 9752 | C | T | 0.538 |
| L2131 | 10463 | T | C | 0.828 |
| L2131 | 11251 | A | G | 0.852 |
| L2131 | 11719 | G | A | 0.974 |
| L2131 | 11812 | A | G | 0.776 |
| L2131 | 12952 | G | C | 0.856 |
| L2131 | 13368 | G | A | 0.954 |
| L2131 | 14233 | A | G | 0.901 |
| L2131 | 14766 | C | T | 0.951 |
| L2131 | 14905 | G | A | 0.99 |
| L2131 | 15326 | A | G | 0.924 |
| L2131 | 15452 | C | A | 0.938 |
| L2131 | 15607 | A | G | 0.76 |
| L2131 | 15928 | G | A | 0.881 |
| L2131 | 16126 | T | C | 0.979 |
| L2131 | 16294 | C | T | 0.872 |
| L2131 | 16304 | T | C | 0.933 |
| L2131 | 16519 | T | C | 1 |
| L2132 | 73 | A | G | 0.967 |
| L2132 | 263 | A | G | 0.923 |
| L2132 | 709 | G | A | 0.901 |
| L2132 | 750 | A | G | 0.722 |
| L2132 | 756 | C | T | 0.463 |
| L2132 | 906 | C | T | 0.679 |
| L2132 | 930 | G | A | 0.891 |
| L2132 | 1147 | G | A | 0.689 |
| L2132 | 1303 | G | A | 0.63 |
| L2132 | 1438 | A | G | 0.695 |
| L2132 | 1489 | G | A | 0.747 |
| L2132 | 1626 | C | T | 0.595 |
| L2132 | 1653 | T | A | 0.8 |
| L2132 | 1676 | A | C | 0.723 |
| L2132 | 1699 | C | A | 0.434 |
| L2132 | 1859 | A | T | 0.76 |
| L2132 | 1888 | G | A | 0.928 |
| L2132 | 1906 | G | A | 0.515 |
| L2132 | 2172 | A | T | 0.741 |
| L2132 | 2557 | C | T | 0.478 |
| L2132 | 2706 | A | G | 0.6 |
| L2132 | 2831 | G | T | 0.75 |
| L2132 | 2906 | C | T | 0.556 |
| L2132 | 3107 | N | T | 0.692 |
| L2132 | 3196 | G | A | 0.672 |
| L2132 | 3525 | C | T | 0.538 |
| L2132 | 3666 | G | A | 0.779 |
| L2132 | 3711 | A | T | 0.848 |
| L2132 | 3900 | C | T | 0.312 |
| L2132 | 3966 | C | T | 0.552 |
| L2132 | 4102 | C | T | 0.325 |
| L2132 | 4216 | T | C | 0.783 |
| L2132 | 4769 | A | G | 0.91 |
| L2132 | 4897 | A | T | 0.883 |
| L2132 | 4917 | A | G | 0.832 |
| L2132 | 5053 | C | T | 0.357 |
| L2132 | 5147 | G | A | 1 |
| L2132 | 5237 | G | A | 0.667 |
| L2132 | 5238 | C | T | 0.6 |
| L2132 | 5703 | G | A | 0.591 |
| L2132 | 6221 | T | C | 0.696 |
| L2132 | 6581 | A | G | 0.873 |
| L2132 | 7028 | C | T | 0.829 |
| L2132 | 8152 | G | A | 0.714 |
| L2132 | 8697 | G | A | 0.969 |
| L2132 | 8860 | A | G | 0.868 |
| L2132 | 10463 | T | C | 0.823 |
| L2132 | 11251 | A | G | 0.84 |
| L2132 | 11653 | A | G | 0.927 |
| L2132 | 11719 | G | A | 0.98 |
| L2132 | 11812 | A | G | 0.795 |
| L2132 | 11852 | G | A | 0.722 |
| L2132 | 12528 | G | A | 0.514 |
| L2132 | 12952 | G | C | 0.831 |
| L2132 | 13368 | G | A | 0.972 |
| L2132 | 14233 | A | G | 0.891 |
| L2132 | 14569 | G | A | 0.571 |
| L2132 | 14766 | C | T | 0.789 |
| L2132 | 14905 | G | A | 0.904 |
| L2132 | 15326 | A | G | 0.933 |
| L2132 | 15452 | C | A | 0.894 |
| L2132 | 15607 | A | G | 0.722 |
| L2132 | 15928 | G | A | 0.875 |
| L2132 | 16126 | T | C | 0.982 |
| L2132 | 16294 | C | T | 1 |
| L2132 | 16296 | C | T | 0.962 |
| L2132 | 16304 | T | C | 1 |
| L2132 | 16519 | T | C | 0.985 |
| L2135 | 263 | A | G | 1 |
| L2135 | 624 | C | T | 0.702 |
| L2135 | 750 | A | G | 0.704 |
| L2135 | 1438 | A | G | 0.732 |
| L2135 | 1515 | G | T | 0.709 |
| L2135 | 1604 | G | A | 0.764 |
| L2135 | 1651 | A | T | 0.704 |
| L2135 | 1658 | T | C | 0.649 |
| L2135 | 1664 | G | A | 0.516 |
| L2135 | 1906 | G | A | 0.537 |
| L2135 | 2172 | A | T | 0.7 |
| L2135 | 2625 | C | T | 0.308 |
| L2135 | 2706 | A | G | 0.633 |
| L2135 | 2831 | G | T | 0.805 |
| L2135 | 2846 | G | A | 0.734 |
| L2135 | 3107 | N | T | 0.875 |
| L2135 | 3194 | T | A | 0.75 |
| L2135 | 3312 | C | T | 0.753 |
| L2135 | 3316 | G | A | 0.676 |
| L2135 | 3462 | C | A | 0.714 |
| L2135 | 3483 | G | A | 0.766 |
| L2135 | 3525 | C | T | 0.529 |
| L2135 | 3633 | T | A | 0.673 |
| L2135 | 3666 | G | A | 0.777 |
| L2135 | 3966 | C | T | 0.6 |
| L2135 | 4062 | T | A | 0.651 |
| L2135 | 4204 | T | C | 0.702 |
| L2135 | 4440 | G | A | 0.696 |
| L2135 | 4643 | G | A | 0.6 |
| L2135 | 4769 | A | G | 0.863 |
| L2135 | 4897 | A | T | 0.909 |
| L2135 | 4898 | C | T | 0.368 |
| L2135 | 4924 | G | A | 0.658 |
| L2135 | 5237 | G | A | 0.741 |
| L2135 | 5238 | C | T | 0.478 |
| L2135 | 5474 | A | C | 0.435 |
| L2135 | 5492 | T | A | 0.5 |
| L2135 | 5581 | A | G | 0.784 |
| L2135 | 5631 | G | A | 0.789 |
| L2135 | 5703 | G | A | 0.75 |
| L2135 | 7013 | G | A | 0.806 |
| L2135 | 7028 | C | T | 0.718 |
| L2135 | 7497 | G | A | 0.833 |
| L2135 | 7521 | G | A | 0.772 |
| L2135 | 7805 | G | A | 0.83 |
| L2135 | 8860 | A | G | 0.849 |
| L2135 | 9752 | C | T | 0.714 |
| L2135 | 10181 | C | T | 0.5 |
| L2135 | 10436 | C | T | 0.515 |
| L2135 | 10736 | C | T | 0.725 |
| L2135 | 11852 | G | A | 0.556 |
| L2135 | 12492 | A | T | 0.786 |
| L2135 | 12528 | G | A | 0.5 |
| L2135 | 14159 | C | T | 0.714 |
| L2135 | 14569 | G | A | 0.5 |
| L2135 | 15326 | A | G | 0.917 |
| L2135 | 16311 | T | C | 0.902 |
| L2135 | 16519 | T | C | 1 |
| L3244 | 239 | T | C | 0.963 |
| L3244 | 263 | A | G | 1 |
| L3244 | 750 | A | G | 0.793 |
| L3244 | 1320 | G | A | 0.795 |
| L3244 | 1438 | A | G | 0.785 |
| L3244 | 1604 | G | A | 0.847 |
| L3244 | 1651 | A | T | 0.838 |
| L3244 | 1957 | A | G | 0.833 |
| L3244 | 2778 | T | C | 0.865 |
| L3244 | 3084 | A | G | 0.784 |
| L3244 | 3107 | N | T | 0.7 |
| L3244 | 3666 | G | A | 0.846 |
| L3244 | 3763 | A | G | 0.849 |
| L3244 | 3915 | G | A | 0.846 |
| L3244 | 4127 | G | A | 0.854 |
| L3244 | 4541 | G | A | 0.556 |
| L3244 | 4727 | A | G | 0.847 |
| L3244 | 4769 | A | G | 0.914 |
| L3244 | 4898 | C | T | 0.869 |
| L3244 | 4960 | C | T | 0.851 |
| L3244 | 5237 | G | A | 0.882 |
| L3244 | 5238 | C | T | 0.8 |
| L3244 | 5492 | T | A | 0.571 |
| L3244 | 5785 | T | C | 0.843 |
| L3244 | 5984 | A | G | 0.878 |
| L3244 | 8860 | A | G | 0.954 |
| L3244 | 9380 | G | A | 0.986 |
| L3244 | 10646 | G | A | 0.94 |
| L3244 | 11253 | T | C | 0.745 |
| L3244 | 11852 | G | A | 0.4 |
| L3244 | 11914 | G | A | 0.982 |
| L3244 | 12528 | G | A | 0.852 |
| L3244 | 12952 | G | C | 0.878 |
| L3244 | 13917 | A | T | 1 |
| L3244 | 15326 | A | G | 0.947 |
| L3244 | 16362 | T | C | 1 |
| L3244 | 16482 | A | G | 0.925 |

| **Year**  **Supplementary Table 3**. Overview of literature search for articles on mitochondrial DNA and Nanopore sequencing. | **Coverage of mtDNA** | **Methyl-ation** | **Tools for methylation calling** | **Sequencing strategy (Kit, Flow cell number, machine)** | **Sample**  **Type (sample size)** | **Disease investigated** | **Enrichment methods** | **Conclusions** | **Reference** |
| --- | --- | --- | --- | --- | --- | --- | --- | --- | --- |
| 2020 | 60.4X | yes | Nanopolish | native mtDNA,  PCR amplicons  (SQK-LSK108, FLO-MIN107, MinION) | Cell line (SAS, H103) | Oral squamous cell carcinoma | Two PCR amplicons for the entire mitochondrial genome (8kb) | - Lower cisplatin sensitivity could be caused by genetic and epigenetic changes of mitochondrial genome | (Aminuddin et al., 2020) |
| 2020 | >10000X | yes | Nanopolish  Guppy + Medaka | native mtDNA  (SGK-RBK004, FLO-MIN106, MinION) | Cell line (HepaRG, HEK293T),  liver tissue | Cancer | Subcellular fractionation, targeted nanopore sequencing | - Low-level of strand specific CpG methylation, higher methylation in tissue compared to cell lines | (Goldsmith et al., 2020) |
| 2020 | 7000X | no | - | CRISPR/Cas9  (SQK-LSK109, FLO-MIN106D, MinION) | Embryonic stem cells (C9012) | - | High Pure PCR template preparation kit | - ONT can detect 11 different thymidine analogs and determine replication rates | (Georgieva et al., 2020) |
| 2019 | 295-813X | no | - | native mtDNA, PCR amplicon  (SQK-RAD001, MKI vR9, MinION) | Blood, cell line (HL-60) | - | PCR amplicon of entire mitochondrial genome | - Direct mtDNA sequencing from native DNA is a reliable alternative to approaches using PCR-enriched libraries | (Zascavage et al., 2019) |
| 2017 | 68X | no | - | native DNA  (NSK007, FLO-MIN106, MinION) | Cell line (HAP1) | - | CsgG-based sequencing | - ONT CsgG-based sequencing may be useful for complex genomes | (Carter and Hussain, 2017) |
| 2020 | NA | no | - | Amplified mtDNA (SQK-LSK109, FLO-MIN106, MinION) | Mouse oocytes, human | NARP/Leigh syndrome | PCR amplicon for the entire mitochondrial genome | - iMiGseq of full mtDNA is good for ultra-senstive variant detection, complete haplotyping and unbiased evaluation of heteroplasmy level | (Bi et al., 2020) |
| 2016 | ~200X | no | - | PCR amplicon (-, FLO-MAP003, MinION) | Blood | - | 2 PCR amplicons for the entire mitochondrial genome | - Hybrid assembly of MiSeq and MinION can accurately recontruct full mitochondrial genomes | (Lindberg et al., 2016) |
| 2021 | 24X (fragmentation)  131X  (BamHI restriction) | yes | Nanopolish | Native mtDNA (SQK-LSK109, EXP-NBD114, FLO-MINI106, MinION) | Human and cancer cell lines | Cancer | Restriction with BamHI-HF in CutSmart buffer (NEB), filtering reads (4-17kb) | - Low level methylaton of mtDNA; Nanopore has less bias than WGBS | (Bicci et al., 2021) |

1. **References**

Aminuddin, A., Ng, P.Y., Leong, C.O., and Chua, E.W. (2020). Mitochondrial DNA alterations may influence the cisplatin responsiveness of oral squamous cell carcinoma. *Scientific Reports* 10.

Bi, C., Wang, L., Fan, Y., Ramos-Mandujano, G., Yuan, B., Zhou, X., Wang, J., Shao, Y., Zhang, P.-Y., Huang, Y., Yu, Y., Izpisua Belmonte, J.C., and Li, M. (2020). Single-cell Individual Complete mtDNA Sequencing Uncovers Hidden Mitochondrial Heterogeneity in Human and Mouse Oocytes. *bioRxiv***,** 2020.2012.2028.424537.

Bicci, I., Calabrese, C., Golder, Z.J., Gomez-Duran, A., and Chinnery, P.F. (2021). Oxford Nanopore sequencing-based protocol to detect CpG methylation in human mitochondrial DNA. *bioRxiv***,** 2021.2002.2020.432086.

Carter, J.M., and Hussain, S. (2017). Robust long-read native DNA sequencing using the ONT CsgG Nanopore system. *Wellcome Open Res* 2**,** 23.

Georgieva, D., Liu, Q., Wang, K., and Egli, D. (2020). Detection of base analogs incorporated during DNA replication by nanopore sequencing. *Nucleic Acids Research* 48.

Goldsmith, C., Rodríguez-Aguilera, J.R., El-Rifai, I., Jarretier, A., Hervieu, V., De Sánchez, V.C., Dante, R., Ichim, G., and Hernandez-Vargas, H. (2020). Minimal detection and low biological fluctuation of mitochondrial CpG methylation at the single-molecule level. *bioRxiv***,** 2020.2009.2014.296269.

Lindberg, M.R., Schmedes, S.E., Hewitt, F.C., Haas, J.L., Ternus, K.L., Kadavy, D.R., and Budowle, B. (2016). A Comparison and Integration of MiSeq and MinION Platforms for Sequencing Single Source and Mixed Mitochondrial Genomes. *PLoS One* 11**,** e0167600.

Zascavage, R.R., Thorson, K., and Planz, J.V. (2019). Nanopore sequencing: An enrichment-free alternative to mitochondrial DNA sequencing. *Electrophoresis* 40**,** 272-280.
